# Supplementary material for: Are carbon emissions trading and green financial instruments synergistic? -Comprehensive quantitative research based on content analysis
Source: PLoS One. 2024 Mar 7;19(3):e0298601. doi: 10.1371/journal.pone.0298601 (PMC10919601; doi:10.1371/journal.pone.0298601)
Supplement: S1 Data — (ZIP) [file pone.0298601.s001.zip › Data/地级市Z_F工作报告.zip!/╜¡╬≈/╕o╓▌╩╨/└┤╫╘╣1⁄2╓┌║┼í╛┬φ┐╦╩2╛▌═°í┐_2014.docx]

各位代表：

现在，我代表市人民政府向大会报告工作，请予审议，并请各位市政协委员和列席会议的同志提出意见。

一、2013年工作回顾

2013年，面对严峻复杂的宏观经济形势，全市上下在省委、省政府和市委的坚强领导下，紧紧围绕建设“幸福抚州”目标，求真务实，锐意进取，经济社会发展有了新的进步。

一年来，我们深入开展“三大战役攻坚年”活动，实现了稳中有进、稳中提质。

经济平稳较快增长。完成生产总值940.64亿元，增长10.3%；财政总收入130.6亿元，增长16.3%，其中公共财政预算收入100.5亿元，增长15.2%；规模以上工业增加值285.99亿元，增长13.4%；社会消费品零售总额339.6亿元，增长12.9%；固定资产投资794.1亿元，增长20.1%；实际利用外资2.91亿美元，增长8.6%；外贸出口12.47亿美元，增长19.2%；城镇居民人均可支配收入20873元，增长10.3%；农民人均纯收入9059元，增长11.9%。生产总值和财政总收入在全省排名双双前进1位，税收收入、公共财政预算收入和县(区)财政收入均突破百亿元大关。

发展质量明显提升。产业结构进一步优化，二、三产业比重比上年提高1个百分点。财税质量继续提升，税收收入占财政总收入比重达到81.8%，在全省排名前进1位，首次超全省平均水平。工业加速崛起，规模以上工业增加值、工业用电量增速均列全省第2；工业对经济增长贡献率达到55.7%，拉动经济增长5.7个百分点；工业税收32.5亿元、增长21.5%，占税收收入30.5%，首次成为第一大支柱税源。建筑业实现产值218.8亿元，增长57.7%。农业稳产提质，粮食总产量286万吨，实现“十连丰”；南丰蜜桔总产量150万吨，再创历史新高；休闲农业实现收入15亿元。服务业加快发展，旅游产业基本实现“县县有国家A级旅游景区”目标，名人雕塑园成为市中心城区首个国家4A级旅游景区；金融机构各项存款余额1111.24亿元，各项贷款余款599.08亿元，增速达18.5%和26.2%，均为全省第一。

发展后劲大为增强。新引进5000万元以上工业项目335个，项目个数和项目增幅均居全省第三。抚州高新区扩区调区工作通过省政府批复，总体规划面积由1333.33公顷扩大到2148.59公顷。抚州电厂获准正式开工建设，向莆铁路正式通车运营，江铃轻汽首辆“骐铃”皮卡下线并完成整车生产1000辆，继“高速梦”之后，再圆“电厂梦”“汽车梦”和“铁路梦”；抚州—金溪、资溪花山界—里木、南昌—乐安—宁都等高速公路开工建设；启动抚州机场项目前期工作；与南昌市、莆田市签订战略合作框架协议，“融入省会、对接海西”迈出重要一步。正式设立江西出入境检验检疫局抚州办事处，开放型经济平台建设实现新的突破。争取到广昌、乐安、黎川、南丰、宜黄、南城、崇仁、金溪、资溪9县列入原中央苏区振兴发展规划范围，享受西部大开发中央预算内投资政策；广昌、黎川、南丰列入《赣闽粤原中央苏区振兴发展规划》核心圈范围。建设用地报批、低丘缓坡综合利用、增减挂总规模达到近4万亩。完成电力基础设施投资6.2亿元，占全省的13%。

一年来，我们坚持建管并重，城乡面貌有了明显改善。

城镇化水平进一步提高。市中心城区实施89个“156”惠民工程项目，完成投资83亿元；各县(区)共实施424个重点城建项目，完成投资176亿元；全市城镇化率达到42.09%，比上年提高1.38个百分点。“四城同创”喜见成效，成功创建“国家园林城市”，荣获我市第一张国家级城市名片。“城市管理年”活动深入开展，全面启动市中心城区市、区、街办、社区四级城市管理体制，对影响市容市貌的顽症痼疾进行了集中整治。

农村生产生活条件进一步改善。投入4.72亿元，建设682个新农村建设村点，完成改水改厕3万余户，硬化道路740公里。实施新农村建设村点和20个乡(镇)集镇农村清洁工程，配备保洁员1491人。推进和谐秀美乡村建设，完成危旧房改造4123栋和绿化植树26.6万株。新建农村公路1441公里。实施水利项目616个，完成投资22亿元，项目个数和投资规模均创历史新高。

一年来，我们在加快发展中注重保护生态，人与自然相处更加和谐。

重点污染源整治强力推进。全市共查处工业企业违法排污案件167件，依法关闭污染大、群众反映强烈的违法排污企业5家。启动了水库养殖和禽畜养殖污染专项整治，终止、协调退出及规范已承包养殖水库970座，完成规模养殖场粪污治理改造592家。开展了凤岗河、梦湖水体污染和农村重点污染区域专项治理，实施了市城区高污染燃料禁燃区管理。

环境质量保持良好。完成了省政府下达的节能减排任务，市辖区地表水12个监测断面水质优良，集中式饮用水水源地水质达标率100%，市城区空气质量优良天数363天，在全省名列前茅。

生态建设成效显著。抚河源纳入国家生态补偿试点范围；完成造林面积33.78万亩，“森林城乡、绿色通道”工作获省政府表彰；封山育林面积达到1234万亩，封山育林工作走在全省前列；成功创建9个国家级生态乡(镇)、10个省级生态乡(镇)和13个省级生态村，建成25个森林乡(镇)和137个森林乡村。

一年来，我们不断加大改革创新力度，经济社会发展增添新的动力。

重点领域改革深入推进。我市作为全省唯一的设区市，被列入全国30个国家中小学教育质量综合评价改革实验区，并成为全省首个省级基础教育综合改革试验区。全国公立医院改革试点单位南城县人民医院综合改革成效明显。完成了国有林场改革前期工作。

创新能力不断增强。科技进步考核综合排序全省第一，荣获全国科技进步先进市称号。“抚州国家精细化工高新技术产业化基地”获批，实现我市国家级产业基地零的突破。发起组建了总规模2.5亿元的江西久富生物医药创业投资基金，并获国家委托投资资金5000万元和省战略性新兴产业投资引导资金2000万元。南丰蜜桔气象指数保险获中国保险产品创新大奖。

一年来，我们着力加强社会建设，“幸福抚州”基础进一步夯实。

民生项目建设扎实推进。全面完成了省政府下达的民生工程任务，民生支出占财政总支出的比重达到67.9%，提高了5.1个百分点；城镇新增就业5.5万人，新增转移农村劳动力5.8万人，城镇就业率达94.6%以上；新开工廉租房和公租房14556套，城市棚户区改造1534户，发放廉租房租赁补贴22147户、3101万元；新农合参合人数增加4.83万人，参合率达到98.99%；贫困人口从27.8万人减少到22万人。市城区新投入运营47辆天然气和60辆油电混合动力公交车，延伸、新开通13条公交线路；实施市中心城区小街小巷道路、路灯、环卫设施新建和改造工程，建设了牛角湾等一批农贸市场，老城区人口密集区建设了一批便民菜店。

社会事业全面发展。教育的财政投入增幅达到13.54%，安排教育重点项目32个，新建中小学校和幼儿园50余所，增加学位4万多个，高考成绩连续十年保持全省第一，录取北大清华的学生人数突破全省的三分之一；成功举办首届“汤显祖戏剧奖·小戏小品奖”大赛和国际环梦湖山地自行车赛。文化新闻出版、广播影视、人口和计划生育、质量技术监督、食品药品监督、国资监管、工商行政管理、民兵预备役、国防动员、人民防空、民族宗教、外事侨务、涉台事务、审计、统计调查、防震减灾、气象、档案、地方志、妇女儿童、老龄、残疾人、红十字会、驻外办事处等工作有了新的进步。

社会保持和谐稳定。积极推进普法宣传教育，深入开展了法律法规常识进乡村、社区、企业等“法律六进”活动。强化风险评估机制建设，对216个建设项目进行了重大事项社会稳定风险评估。切实加强和创新群众工作，创新并推广了农村理事会、“和事厅”等依靠群众预防和化解社会矛盾的做法。始终保持对各种犯罪活动的高压严打态势，开展了社会治安集中整治“百日行动”、破案会战、夏季行动、打黑除恶等系列专项行动。完善治安防控体系，在全省率先完成了“天网”工程三期建设。全市社会形势呈现平稳可控、持续向好的发展态势，为建设“幸福抚州”提供了稳定的社会环境和法治环境。

各位代表，在宏观形势错综复杂、经济下行压力加大的情况下，全市经济社会呈现出快速健康协调发展的好势头，这是认真贯彻党中央、国务院和省委、省政府一系列重大决策部署的结果，是市委正确领导的结果，是市人大、市政协监督支持的结果，是全市上下凝心聚力、真抓实干的结果。在此，我谨代表市人民政府，向全市广大干部群众，向各民主党派、工商联、无党派、人民团体、社会各界人士，向中央、省驻抚单位，向驻抚部队和武警、消防官兵，向外来投资者和建设者，向所有关心、支持和帮助抚州发展的同志们、朋友们，表示衷心的感谢和崇高的敬意!

同时，我们也清醒地认识到发展中存在的困难和问题，主要是：经济总量偏小；支柱产业不强；财政收支矛盾突出；社会稳定的基础尚不牢固；干部攻坚克难的能力有待加强等。对此，我们将高度重视，在下一步工作中切实加以解决。

二、2014年工作安排

今年是深入贯彻党的十八届三中全会精神、全面深化改革的开局之年，也是“三大战役”提升之年，做好政府各项工作，意义重大，影响深远。

今年政府工作的总体要求是：全面贯彻落实党的十八大、十八届三中全会、中央经济工作会议和省委十三届七次、八次全会精神，紧紧围绕市第三次党代会的总体部署，牢牢把握稳中求进、改革创新总基调，稳增长，提质量，抓改革，优生态，强作风，惠民生，以开展党的群众路线教育实践活动为动力，以开展“三大战役提升年”活动为总抓手，统筹做好各项工作，为建设殷实、文明、和谐的“幸福抚州”打下坚实基础。

今年全市经济发展的主要预期目标是：生产总值增长10%，达到1060亿元；财政总收入增长13%，达到147亿元，力争增长15%，突破150亿元；规模以上工业增加值增长15%，达到328亿元；固定资产投资增长20%，达到952亿元；社会消费品零售总额增长13.5%，达到385亿元；出口总额增长8%，达到13.5亿美元；实际利用外资增长10%，达到3.2亿美元；城镇居民人均可支配收入增长10%，达到22960元，农民人均纯收入增长11%，突破10000元。

根据上述目标要求，今年重点做好八个方面的工作：

(一)全面深化改革，充分激发发展活力

积极创新区域发展战略。紧扣“一条主线”，即“融入省会、对接海西”，努力争取建设赣闽开放合作创新区上升为省级发展战略，形成“一化两园一带”产业格局。继续抓好“三区一化”战略实施，争取《赣闽粤原中央苏区振兴发展规划》涉及我市的政策、事项、项目落地；加强与中央国家机关的汇报与沟通，科学编制对口支援规划方案，确保对口支援工作取得实效。

切实转变政府职能。深化政府机构改革，三季度前全面完成市、县(区)政府机构改革。深化行政审批制度改革，全面推行“负面清单”制，各职能部门重新梳理上报行政审批事项，列入政府目录清单管理，向社会公布，目录清单以外的一律取消审批。研究探索“多证(照)一体”改革，更大限度精简办事流程、提高办事效率。加快政务服务平台建设，积极推进网上审批、网上交易、网络问政、网上监察实质性融合，提升审批服务效能。积极推动向社会组织转移职能，探索“资格资质脱政化”改革，逐步建立健全政府购买服务机制。

不断完善公平竞争的市场体系。打破地区之间、行业之间、所有制之间的市场壁垒，实行统一的市场准入和市场监管制度，对各类市场主体一视同仁。坚持“非禁即入”原则，取消和废止制约民间投资的各种歧视性政策，重点在交通运输、公共基础设施建设、教育卫生、文化体育和旅游、金融等领域进一步放开放活，鼓励民间资本深度参与科技、水利、通信、电力、环保、社会福利等项目建设。完善市场体系，实现商品和要素自由流动、平等交换。积极推进工商登记制度改革，实行“先照后证”和注册资本认缴登记制。

加快创新国有资本运营机制。以理顺经营体制、实施政企分开、资产保值增值、公平参与竞争为重点，推动市投资发展(集团)有限公司市场化运作、实体化经营、规范化管理。鼓励建筑施工、工程技术、规划设计等企事业单位改制，发展混合所有制经济。做好国有企业改制扫尾工作，解决好历史遗留问题。建立组织选拔和市场化选聘相结合的用人制度，逐步加大市场化选聘比例，加快建设职业经理人队伍。

稳步推进事业单位分类改革。加快事业单位管办分离，完善法人治理结构，推动社会力量兴办公益事业。继续推进职能相近的事业单位整合工作，重点整合公共资源交易服务机构和业务相近的检验、检测、认证机构。深化食品安全体制改革和医药卫生改革，统筹推进事业单位分类改革与机构清理、机关事业单位超编专项整治和在编不在岗人员清理工作。

加快推进农村土地承包经营权流转。鼓励和支持农村土地向专业大户、家庭农场、农民专业合作社流转，发展多种形式的适度规模经营。大力倡导以股权为纽带的农村土地流转，鼓励、引导农户以农村土地承包经营权入股的形式组建农民专业合作社。选择1—2个县(区)启动农村土地承包经营权流转交易市场试点，鼓励其他有条件的地方同步开展试点，逐步建立市、县、乡三级农村土地承包经营权流转交易市场。

(二)坚定不移地强攻工业，加快推动发展升级

加快产业集聚。按照“工业一县一业”的思路，培育发展一批重点产业集群。坚持特色化引领，提升、打造高新区汽车零部件和生物医药、金溪香料、宜黄塑料、崇仁变电设备、黎川日用陶瓷、临川有色金属加工、东乡医药化工、南城南丰广昌绿色食品加工等一批具有比较优势，“有根”“有据”的特色产业集群；坚持区域化布局，编制完善产业集群发展规划，优化要素资源配置，探索招商引资利益共享机制，引导同类企业加快集聚；坚持差异化扶持，研究制定优惠和倾斜的政策措施，创造有利于产业集群发展的良好环境；坚持品牌化建设，实施区域品牌战略和企业品牌战略，提升集群和企业的核心竞争力；坚持链条化发展，推动产业集群与专业化市场互动发展，鼓励有条件的产业集群发展工业旅游、产业旅游等。力争六大支柱产业主营业务收入达到1450亿元，其中机电汽车、化工建材产业分别过300亿元，有色金属加工突破400亿元。

培育企业集群。做大做强龙头企业，辐射带动中小企业，努力形成“大企业顶天立地、中小企业铺天盖地”的生动局面。确定一批重点扶持的工业企业，集中各种资源要素，帮助其技改扩能，转型升级，促进做大做强，裂变式发展。重点推进高特佳等新开项目建设，加快建设广银铝业、江铃轻汽、金品铜业、飞达钢业等企业续建项目，抓好珍视明药业、科伦药业、银涛药业、圣农食品、自立铜业等企业竣工项目达产达标，支持江铃底盘、苍源药业、洪门实业、施美制药等企业股改上市或到“新三板”挂牌融资。力争规模以上工业企业达900户以上，完成主营业务收入突破1600亿元，工业投资占全社会固定资产投资比重达到65%。

促进园区集约。鼓励企业建设多层厂房，将园区准入门槛提高到单位面积投资200万元/亩、主营业务收入300万元/亩、税收5万元/亩、容积率1.0以上。在全市范围内开展园区用地集中清理行动，依法依规对低效用地、闲置土地进行清理、收回。抓紧做好9个获准开展前期工作的省级工业园区的规划编制和材料申报工作。完善园区道路、水电、燃气、通信管网等基础设施，争取年内建成220千伏抚州城南输变电工程等一批重点电网项目。加大园区“办社会”力度，抓好“六个一”项目的建设、运转。努力争取高新区升级为国家级高新技术产业园区，积极争取南城等工业园区申报省级重点工业园区，乐安工业园区列入省级工业园区。力争年内新增主营业务收入过100亿元和过200亿元的园区各1个，抚州高新区争取跨入400亿园区行列。

提升服务水平。积极搭建五个服务平台，一是供需协作平台，进一步将企业产品原材料供需对接活动常态化、制度化。二是融资平台，积极开展政银企对接活动，确保全年新增工业贷款高于全市各项贷款平均增速5个百分点以上；做大做强融资担保机构，扩大融资担保业务规模；推进企业用好用活股权融资、债务融资等工具，提高直接融资比例。三是科技创新平台，推进科技协同创新，力争全市高新技术产业产值增长20%以上。四是招工用工平台，推动职业技术教育院校与园区有效对接，继续开展“欢乐春节家乡行”等用工对接活动，在各工业园区设立常年的招工窗口。五是创业孵化平台，加快小企业创业基地建设步伐，组织开展创业人员培训。同时进一步强化重点企业特派员制度和领导挂点帮扶制度。

(三)有序推进新型城镇化，更好地统筹城乡发展

推进以人为核心的城镇化。加快从空间城镇化向人口城镇化转变，促进农业转移人口市民化，按照中央部署，积极推进农村和城市社会保障体系并轨，逐步解决进城务工人员子女平等接受义务教育问题。推进城镇户籍管理制度改革，积极放开市中心城区落户限制，全面放开县城和乡(镇)落户限制。抓紧制定与户籍制度改革相配套的政策和措施，在维护农民基本权益的前提下，积极探索农业转移人口市民化成本分担机制。加大进城农民的创业就业扶持力度，努力提高农民工融入城镇的素质和能力。

建立新型城镇化体系。以新一轮城市总体规划修编为契机，整合国民经济与社会发展规划、城市总体规划、主体功能区规划、土地利用总体规划、生态建设规划等内容，形成“五规融合”的城乡规划体系。启动新一轮城镇化建设，构建以市中心城区为龙头、县城为骨干、集镇为补充的新型城镇体系。抓紧启动行政区划调整工作，着力推动增设市辖区，鼓励条件成熟的地方撤县改市，推进已融合到市中心城区的乡(镇)改设街道办事处，同时优化整合现有街道办事处设置，合理调整街道、社区管辖范围，鼓励具备条件的乡改镇。

建设宜居城镇。加快完善城市公交、道路、给水、绿化、排污等基础设施和文化、教育、医疗卫生、金融、商业等服务设施，重点推进100个市级重大项目建设，着力实施一批县(区)和乡(镇)重点城建项目。建立多元可持续的资金保障机制，力争市投资发展(集团)有限公司等融资平台完成融资到位资金40亿元。进一步完善市中心城区“两级政府、三级管理、四级网络”的城市管理体制，建立健全乡(镇)规划建设、环卫管理体制。深入推进“四城同创”，力争年内成功创建“国家森林城市”和“省级卫生城市”。深化市中心城区交通秩序、客运市场和砂土运输专项整治，开展乡(镇)环境综合整治行动。启动“智慧抚州”总体规划编制工作，积极申报智慧城市建设试点，推进“数字档案馆”工程建设，提升城市信息化管理水平。

促进城乡一体化发展。强化县域在城乡一体化中的主导作用，深入开展县域经济发展大会战。推进镇村联动，重点抓好城关镇、中心镇和中心村建设，推进产业向规划区集中、农户向社区集中、农业用地向规模经营集中，加快消除城乡二元结构，实现城乡规划衔接、设施对接、产业联动、资源共享，构建城乡经济社会一体化新格局。扎实打好扶贫攻坚战，促进区域协调发展。

(四)扎实推进农业农村现代化，稳步提高“三农”工作水平

发展现代农业。高度重视粮食安全，深入推进“粮食稳产增产十大行动”，确保粮食播种面积稳定在610万亩以上，总产达到55亿斤以上。大力推进农业机械化，力争农机总动力达到600万千瓦。按照“以县为主、一县一品，整体规划、分步实施，统一管理、市场运作”原则，全力推动11个规划面积不少于1万亩、核心区不少于5000亩的现代特色农业示范园建设。深入推进农业标准化工程，力争新增2个国家级农业标准化示范区。依托特色农产品基地、自然风景名胜和民俗风情等资源优势，打造各具特色的休闲农业精品，延伸农业价值链。抓好去冬今春水利工程建设项目扫尾和验收工作，同时实施277个水利项目，完成投资7亿元。做好廖坊灌区一期验收和二期前期工作，抓好廖坊灌区生态农业示范区试点建设。

培育新型农业经营主体。实施“强龙增值富民”工程，大力扶持产业化龙头企业、家庭农场、专业大户、农民专业合作社等新型主体，引导生产要素加快集聚，争取全市市级以上农业产业化龙头企业突破400家，实现主营业务收入240亿元，其中年销售收入超5亿元5家，超亿元45家；支持龙头企业领办农民专业合作社，建立完善“公司+农民专业合作社+农户”的利益联接机制，整合涉农项目资金并向合作社倾斜，确保全年新增农民专业合作社200家；积极推进职业农民队伍建设，同时继续重视普通农户的发展。

建设农村美丽家园。以镇村联动、村落连片、中心村和交通主干道沿线村庄整治为重点，力争安排50户以上新农村建设村点700个左右，每个县(区)至少抓好1个集镇、1个中心村和1个200户以上大村的整治，形成集中连片示范效应。大力实施农村生态环境整治，扎实推进农村清洁工程，加快农村危旧房改造，抓好农业节能减排。打造向莆铁路沿线和谐秀美乡村，重点推进向莆铁路沿线5个县(区)、83个行政村、227个自然村的和谐秀美乡村建设。

(五)大力发展现代服务业，着力培育新的经济增长极

大力发展旅游业。实施旅游强市战略，加快组建市旅游投资集团并力争融资10亿元以上，确保全市今年旅游投入总规模不少于100亿元。抓好王安石文化公园、文昌里、灵谷峰、临川温泉、乐安流坑、资溪九龙湖、大觉山二期、方家山景区、南丰桔园休闲游、军峰山、南城洪门湖和麻姑山景区、黎川古城、宜黄曹山寺、金溪浒湾古镇、广昌驿前姚西景区、崇仁源野山庄和汤溪泉城、东乡佛岭公园等重点景区建设，着力打造一批特色鲜明、风格独特、吸引力和竞争力较强的旅游景区。积极推进大觉山景区创评国家5A级、南城洪门湖景区创评国家4A级旅游景区，支持资溪县开展旅游综合改革试点。

大力发展现代物流业。抓好抚北综合物流园、抚州市农产品配送中心和各县(区)物流中心建设，加快形成市中心城区为核心，东乡、崇仁、广昌、南城、南丰为联络节点的5大物流集聚区。借助向莆铁路通江达海优势，大力发展多式联运，力争开通赣闽“五定”班列。大力引进和培育第三方物流龙头企业，提高物流专业化、信息化水平。发挥市物流协会作用，抓住“营改增”税收政策调整机遇，促进本土物流企业优化整合，提升品质，规范发展。努力将我市打造成为闽赣乃至中部地区物流集散地。

大力发展现代商贸业。尽快编制完成城市商业网点规划，进一步优化商业空间布局。推动建设赣东国际汽车城、赣东农机交易市场、水果批发市场、生猪批发市场等专业市场，加快建设华美立家家居建材市场、仙峰商业中心等一批城市综合体项目，不断提升市、县城区特别是市中心城区的商贸吸引力。深入推进“万村千乡”市场工程，完善城乡一体的市场体系。大力发展电子商务，支持鼓励新办电子商务企业，引进电子商务总部，创办电子商务园区。大力发展总部经济，加快建设总部经济中心并引进企业入驻。努力将我市打造成为赣东区域现代商贸中心。

大力发展现代金融业。争取昌抚金融同城化，启动民间融资服务中心试点工作，引导并规范银行加强与证券、期货、基金、信托等非银行金融机构的合作。运作好江西久富生物医药创业投资基金，力争成功发行1支“区域集优”票据。力争新引进1家以上银行来抚设点，新增设立1家以上村镇银行，新申请开办3家以上小额贷款公司。深化农村信用社改革，力争组建2家县级农村商业银行。力争年内新增存款达到200亿元、新增贷款达到150亿元。大力发展保险市场，充分发挥保险参与经济社会管理功能，积极做好“险资入赣”对接工作。

促进房地产健康发展和建筑业振兴发展。贯彻落实房地产调控政策，促进房地产市场平稳健康有序发展。支持建筑企业联合、兼并、重组，做强做大。落实好支持建筑业发展扶持政策，鼓励市外建筑企业落户。打造抚州建筑总部基地，积极推动抚州建筑企业拓展省外建筑市场，支持中阳、玉茗两家企业晋升特级企业，支持其他二级骨干企业加快资质升级，重振抚州建筑业雄风。力争全市建筑业总产值增长20%以上，对地方财政贡献率增长30%以上。

大力发展家政养老等服务业。建设家政服务网络中心平台，培育一批家政服务企业，推进家政服务市场化、社会化和产业化发展，满足社会家政服务需求，提升从业人员素质和服务质量。研究制定养老事业发展专项规划，加快建立政府引导、社会化服务为主的城乡居家养老和机构养老体系。

(六)进一步扩大对外开放，不断提升区域竞争力

完善开放型经济体系。积极推进抚州电厂、抚北互通及出口连接线、抚州—金溪、资溪花山界—里木、南昌—乐安—宁都高速公路建设。做好鹰梅铁路、吉安—抚州—武夷山铁路、抚州机场和抚河通航工程的前期工作；做好东乡—昌傅、广昌—船顶隘、广昌—吉安高速公路的前期工作，争取年内开工。开工建设江西出入境检验检疫局抚州办事处检测大楼并尽快完工交付使用；积极筹建抚州铁路口岸作业区和公路港，支持南丰建设公路口岸作业区；继续争取设立海关机构；积极争取申报抚州综合保税区或国家级出口加工区。

创新招商方式。改变以往“大兵团”招商和大会招商模式，实现从单个项目招商向产业链招商、分散招商向集中招商转变，市本级和各县(区)分别组建一批专业招商团队，积极开展驻点招商、以商招商、委托招商和网络招商，力争全市新引进5000万元以上工业项目230个，实际进资320亿元以上，其中亿元以上项目115个，实际进资200亿元以上。同时着力提升利用外资水平和扩大外贸出口，力争现汇利用率达到30%以上，生产型出口企业占比达到40%。

大力招才引智。紧紧围绕强攻工业、推进新型城镇化、旅游强市、加快发展现代服务业等重点工作，着力引进城乡规划、旅游、电子商务、金融、教育卫生、农林水等领域的紧缺急需高素质人才；推动技术创新，努力将高新区10万平方米中小企业创业园打造成为全市的高新企业孵化基地、科技成果转化基地、创业就业服务平台、企业与高等院校科研院所对接平台。

(七)积极推进生态文明建设，切实增强可持续发展能力

实施生态保护和生态修复工程。培育保护森林资源，完成造林绿化面积30万亩，重点做好向莆铁路沿线造林绿化，着力实施“一人一桂”绿化工程，加强护林防火和病虫害防治，扩大封山育林面积；大力推进生态县、生态乡(镇)、生态村和生态工业园区建设，力争全市10个省级工业园区全部建成生态工业园区，推进资溪县低碳经济试点工作；加强各自然保护区、风景名胜区、森林公园、湿地公园的建设和保护，做好抚河源国家生态补偿试点工作，支持资溪等县列入武夷山生物多样性功能保护区；稳妥推进垃圾处理无害化、资源化工作。

完善项目准入制度。严格执行项目环境影响评价制度和“三同时”制度，逐步提高项目准入门槛，进一步提高项目质量。实行新建项目环评预审制度，避免企业盲目建设、投资；加大对“未批先建”“久试不验”项目的清理工作，加大整改处罚力度。

加强环境执法。深入开展“整治违法排污企业保障群众健康”专项行动，对污染严重、治理设施不到位的企业实行挂牌督办，限期整改。着力实施主要污染物重点减排工程，加大水库水质污染和畜禽养殖污染专项治理力度，推进污染综合防治和农业面源污染控制，加快实施农村环境综合整治项目，完善生活污水配套管网建设，推动工业园区污水处理厂及配套管网建设。

(八)努力打造安居乐业环境，持续深化建设“幸福抚州”内涵

完善社会保障体系。进一步完善社会保险制度，推进养老保险和医疗保险制度落实。进一步提高城乡低保标准和补差水平，适度扩大全市农村低保覆盖面。开展最低生活保障城乡一体化工作试点。基本实现医疗救助城乡一体化。完善农村五保供养制度，提高集中供养水平，加大农村敬老院建设改造力度，力争从今年起用2—3年时间，每个乡(镇)至少建成一个标准化敬老院。大力实施市中心城区“千百万”棚户区改造工程，基本完成抚纺片区棚户区改造，全面启动文昌里片区棚户区改造。加快推进保障性住房建设，统筹推进棚户区改造与保障性住房建设、重点项目拆迁安置房建设。

统筹发展社会事业。实施教育优先发展战略，启动国家中小学教育质量综合评价改革实验区和抚州基础教育综合改革试验区建设，优化城乡教育资源配置，推动教育均衡协调发展，提升抚州教育品牌。大力实施文化惠民工程，鼓励创作临川文化精品。强化公共卫生服务体系建设，不断提升医疗服务质量。推进质量兴市工作，积极创建省级食品药品安全示范区，打造安全放心的饮食用药环境。大力发展群众体育和竞技体育，推进科技协同创新，加强人口和计划生育、广播影视和新闻出版等工作，加强国防教育和民兵预备役建设，巩固扩大双拥共建成果，支持民主党派、无党派、工商联及人民团体工作。

加强和创新社会治理。坚持系统治理、依法治理、综合治理、源头治理，特别是要善于运用法治思维和法治方式，依法化解社会矛盾纠纷，坚守政策法规底线，促进社会公平正义。加强以基层基础为重点的社会综治队伍建设，逐步加大基层工作经费投入，充分调动广大基层干部的积极性，构建完善的基层平安创建工作体系。深化平安抚州建设和法治抚州建设，进一步健全立体化治安防控体系，提高全民法律素质和全社会法治化管理水平，提高人民群众安全感。狠抓安全生产责任制落实，促进安全生产形势持续稳定好转。改革信访机制，认真做好信访工作，畅通群众诉求表达渠道，倾听群众呼声，关心群众疾苦，切实为群众排忧解难。引导群众依法理性表达诉求，依法处置无理闹访行为，依法严厉打击扰乱公共秩序危害公共利益的行为。确保不发生重大政治影响事件，不发生重大群体性事件，不发生极端恶性事件，不发生大规模集体赴省进京上访事件，不发生严重危害群众安全的重大刑事案件和重大治安问题，不发生重特大安全责任事故，为建设“幸福抚州”营造更加和谐稳定的社会环境。

各位代表!

做好今年的政府工作，打造一支作风过硬、战斗力强的工作团队是前提和基础。新的一年，我们将重点从四方面加强政府自身建设。坚持群众路线，切实转变作风。深入扎实开展好党的群众路线教育实践活动，紧紧围绕“为民、务实、清廉”主题，牢牢把握“照镜子、正衣冠、洗洗澡、治治病”的总要求，聚焦作风建设，从领导干部做起，集中解决“四风”问题，不断提高服务群众的能力和水平。推进依法行政，加快法治化进程。进一步落实好领导干部学法制度，切实增强依法行政的意识和能力。不断加强制度建设，进一步规范行政权力运行。完善科学民主决策机制，坚持重大事项集体决策，健全专家咨询论证、社会公示和听证等制度。自觉接受人大及其常委会监督，充分利用人民政协人才集聚、联系广泛的优势，认真听取各民主党派、无党派、工商联、人民团体和社会各界意见。强化绩效管理，提升行政效能。健全效能考核激励机制，完善绩效考评制度，推进效能建设制度化、规范化、常态化。加强政府效能监察，加大行政问责力度，确保政令畅通和绩效提升。深化整治“庸懒散”行动，坚决查处行政不作为、乱作为及“吃拿卡要”等损害发展环境的行为。完善以科学发展为导向的政绩考核评价体系，引导干部树立正确的政绩观。坚持惩防并举，促进廉洁从政。严格落实党风廉政建设责任制，完善政务公开和各领域办事公开制度，强化对财政资金、重大投资项目的审计和监察，推动源头治理腐败。进一步强化惩治和预防腐败体系建设，健全工程招投标、土地交易、政府采购、资金监管等重点领域的反腐倡廉长效机制，全面落实中央厉行节约反对浪费和规范公务接待管理有关规定，进一步推进“三公”改革。严肃查处违法违纪案件，营造风清气正、廉洁高效的政务环境。

各位代表，“春风又绿江南岸”，党的十八届三中全会吹响了全面深化改革的号角。让我们紧密团结在以习近平同志为总书记的党中央周围，在省委、省政府和市委的坚强领导下，开拓创新，砥砺奋进，不负重托，无愧使命，努力开创建设殷实、文明、和谐的“幸福抚州”新局面!
